# Breeding of a new malting barley variety ‘Satuiku 5 go’ for Hokkaido exhibiting improved grain yield and malting quality

**DOI:** 10.1270/jsbbs.23094

**Published:** 2024-08-27

**Authors:** Yoshiro Tokizono, Satoshi Asayama, Hironobu Jinno, Kazuya Araki, Nana Ashikaga, Ryohei Fujita, Hirotake Ito, Toshihisa Watanabe, Ryoichi Kanatani, Makoto Kihara, Naohiko Hirota, Masahito Nanamori, Hozumi Yoshida, Takehiro Hoki

**Affiliations:** 1 Crop Research Laboratories, Sapporo Breweries Ltd., 37-1 Nittakizaki, Ota, Gunma 370-0321, Japan; 2 Kitami Agricultural Experiment Station, Hokkaido Research Organization, 52 Yayoi, Kunneppu, Tokoro, Hokkaido 099-1496, Japan; 3 Kamikawa Agricultural Experiment Station, Hokkaido Research Organization, 1-5 South, Pippu, Kamikawa, Hokkaido 078-0311, Japan; 4 Tokyo University of Agriculture, 196 Yasaka, Abashiri, Hokkaido 099-2493, Japan; 5 Product & Technology Innovation Department, Sapporo Breweries Ltd., 10, Okatome, Yaizu, Shizuoka 425-0013, Japan

**Keywords:** malting barley, lipoxygenase-1, high yield, beer flavor stability, Hokkaido, Satuiku 5 go

## Abstract

Hokkaido-specific malting barley varieties have been developed to improve the grain yield, disease resistance, malting quality, and brewing quality. In this report we describe the breeding and evaluation of brewing quality of a hulled two-row malting barley (*Hordeum vulgare* L.) variety ‘Satuiku 5 go’ lacking lipoxygenase-1 (LOX-1-less). ‘Satuiku 5 go’ was evaluated in the joint field trials for malting barley in Hokkaido from 2016 to 2018. ‘Satuiku 5 go’ exhibited 11% higher grain yield and 7.6 cm shorter plant height than the control variety, ‘Ryohfu’. However, the disease severity of Fusarium head blight (FHB) in ‘Satuiku 5 go’ was higher than in ‘Ryohfu’. For malting quality, ‘Satuiku 5 go’ exhibited higher diastatic power, soluble nitrogen content, and fine extract content, and lower wort β-glucan content than ‘Ryohfu’. 100-litter pilot scale brewing trials were conducted with ‘Satuiku 5 go’ and ‘Satuiku 2 go’ as a control variety, also a LOX-1-less variety, and no clear differences were observed.

## Introduction

Malting barley breeding is required to improve all the agronomic traits, malting and brewing quality. Public breeding institutions such as Tochigi Prefectural Agricultural Experimental Station and Fukuoka Agriculture and Forestry Research Center, and Sapporo Breweries Limited are engaged in the breeding and evaluation of the agronomic traits, malting quality, and brewing quality of malting barley in Japan. Tochigi Prefectural Agricultural Experimental Station, Fukuoka Agriculture and Forestry Research Center, and Sapporo Breweries Limited conduct breeding experiments and hold discussions on the agronomic traits, malting and brewing quality with the Brewers Association of Japan, Growers’ group, and national brewing companies as actual consumers. The final approval for commercialization is made by all the parties involved. For Honshu region, the lipoxygenase-1 (LOX-1) null variety ‘New Sachiho Golden’ was developed at the Tochigi Prefectural Agricultural Experimental Station by backcross breeding using the major malting variety ‘Sachiho Golden’ as a recurrent parent (Oozeki *et al.* 2017). ‘Harusayaka’ was developed by Fukuoka Agriculture and Forestry Research Center (Kai *et al.* 2021) for Kyushu region registered a higher yield and showed a lower occurrence rate of skin cracking than ‘Sachiho Golden’. As shown in these examples, several varieties have been developed and put on the market in Honshu in recent years.

In Hokkaido, The Kaitakushi Brewery (The Hokkaido Development Commission Brewery, predecessor of Sapporo Breweries Limited) was involved in breeding and growing malting barley to ensure stable procurement of raw materials for brewing beer in the late 19^th^ century. ‘Ryohfu’ was bred in 1989 by Kitami Agricultural Experiment Station and cultivated as a leading variety for about 30 years. Sapporo Breweries Limited and Kitami Agricultural Experiment Station filed a joint variety application of ‘Hokuiku 39 go’ in 2005 to improve agronomic characteristics and malting quality compared with ‘Ryohfu’. However, ‘Hokuiku 39 go’ was not popularized because of the high *S*-methyl-methionine (SMM) content, which had a negative effect on beer flavor. Afterwards, only Sapporo Breweries Limited conducted malting barley breeding in Hokkaido beginning from 2008 and developed the LOX-1-less barley variety ‘Satuiku 2 go’ in 2016 (Hoki *et al.* 2018). LOX-1 catalyzes the formation of precursors of *trans*-2-nonenal (T2N) and trihydroxyoctadecenoic acid (THOD). T2N is known to account for cardboard-like flavor in stale beer (Drost *et al.* 1990) and THOD is known to affect a negative effect on the smoothness and foam stability of beer (Kaneda *et al.* 2001, Kobayashi *et al.* 2002). ‘Satuiku 2 go’ replaced ‘Ryohfu’ from 2019 and is still grown as a major variety in Hokkaido. However, ‘Satuiku 2 go’ was developed by backcross breeding targeted for LOX-1-less trait with molecular marker-assisted selection (Hirota *et al.* 2005) using ‘Ryohfu’ as the recurrent parent. Consequently, although brewing quality was improved by the introduction of the LOX-1-less trait (Hoki *et al.* 2018), there was no major improvement in grain yield, other agronomic traits and malting quality of malting barley varieties in Hokkaido over the past 30 years.

In order to solve the quality issues in ‘Hokuiku 39 go’ and the yield issues in ‘Satuiku 2 go’, we worked on breeding malting barley coupled with higher yield, better malting and brewing quality, and the LOX-1-less trait. In this paper, we describe breeding of the new malting barley variety ‘Satuiku 5 go’ and the improvement made for agronomic traits, malting quality and brewing performance.

## Materials and Methods

### Breeding process and field trials

**Fig. 1.**
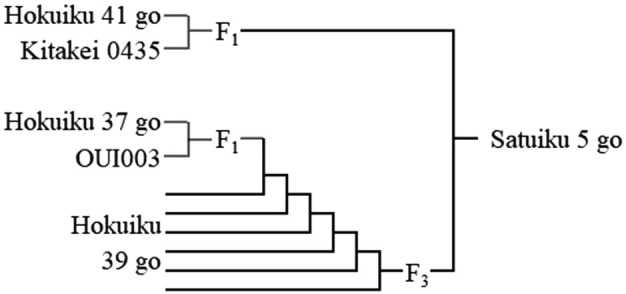
Pedigree of ‘Satuiku 5 go’.

The original cross between the F_1_ plants from the ‘Hokuiku 41 go’/‘Kitakei 0435’ cross and the F_3_ plants from the ‘Hokuiku 37 go’/‘OUI003’//‘Hokuiku 39 go’///5* ‘Hokuiku 39 go’ cross was carried out in a greenhouse at Sapporo Breweries Ltd. in Gunma Prefecture (36°16ʹN, 139°18ʹE) in 2008 (Fig. 1).

In 2008, the F_1_ plants were grown as a bulk population in a summer nursery in Hokkaido (43°55ʹN, 144°23ʹE), which is a spring-sown area. From 2008 to 2009, the F_2_ to F_4_ plants were grown as bulked populations between Hokkaido and New Zealand, using contra-season growing methods. The F_5_ plants were grown in Hokkaido for spike selection in summer 2010. The selected spikes were checked for the LOX-1-less trait using molecular marker-assisted selection and then only selected lines with the LOX-1-less trait were grown as F_6_ line selection in 2011. The selected lines from F_6_ generations were grown for the subsequent selection in 2012 and a selected line from F_7_ lines was named ‘Satukei 1323’. After three years of yield trials from 2013 to 2015, the line was renamed ‘Satuiku 5 go’ and tested in the joint field trials for malting barley in Hokkaido from 2016 to 2018. The trials were conducted at three to five sites in Hokkaido with two to four replicates, in which the layout was completely randomized within each replicate. The plot size of each replicate was 3.0 m^2^. The agronomic performance in these yield trials was observed according to the methods described by Hoki *et al.* (2018). We used two control varieties, ‘Ryohfu’ as an agronomical control in the joint field trials for malting barley and ‘Satuiku 2 go’ for the testing of pilot scale brewing. Due to the transition of commercial varieties mentioned above, we used each variety for those trials to confirm and evaluate the characteristics of ‘Satuiku 5 go’.

### Micro-malting system-based evaluation of malting quality

Harvested grain collected in the joint field trials for malting barley were malted on a microscale (250 g barley grain samples) using an automatic micro-malting machine (Phoenix Biosystems, Adelaide, Australia). Micro-malting and malt analyses were performed as described by Hoki *et al.* (2018). SMM was measured in three to five replications from 2016 to 2018 according to the methods described by Nanamori *et al.* (2011).

### Pilot-scale brewing trial

100-litter pilot scale brewing trials were conducted according to our standard method (Nanamori *et al.* 2021). ‘Satuiku 5 go’ and ‘Satuiku 2 go’ (control) were grown and harvested in 2017 in the test fields at the Abashiri Cold Region Farm, Tokyo University of Agriculture in Hokkaido. Grain samples of each variety were malted using an automatic micro-malting machine (Phoenix Biosystems). Wort was prepared from malts (71%, w/w), starch, corn, rice, and hops using a 100-liter mashing apparatus. Mashing was done for 5 min at 60°C, followed by 35 min at 67°C, and then 5 min at 75°C. The wort was boiled for 90 min and diluted with hot water to a concentration of ca. 11.0% of the extract. After cooling, 15.0 × 10^6^ cells mL^–1^ of lager yeast was added to the wort to start fermentation, which was carried out at 9.0–12.0°C for seven days followed by maturation for eight days at 13.0°C, and then 21 days at 0°C.

### Measurement of trans-2-nonenal (T2N) and trihydroxyoctadecenoic acid (THOD)

T2N and THOD contents were determined using methods described by Hirota *et al.* (2006a, 2006b) and Kobayashi *et al.* (2000), respectively.

### Sensory evaluation

The beers were stored at 37°C for one week and 30°C for one month. We scored the aged beers for total staleness during the sensory evaluation. Total staleness was defined as the overall impression of staleness for off-flavor and stale of beer. Trained panelists evaluated the beers based on a scale of 0 (fresh) to 4 (strongly stale) at 0.5 intervals (Nanamori *et al.* 2021).

### Test of Fusarium head blight (FHB) resistance

Evaluation of FHB resistance was conducted from 2016 to 2018 at the Kitami Agricultural Experimental Station. The test plots were planted in two replicates, and the plot size for each replication was 0.6 m^2^. A spore suspension of *Fusarium graminearum* Schwabe (strain TYK-101) was sprayed and inoculated on spikes five days after the heading in the field trial; a seven-minute-mist spray with sprinklers was carried out hourly throughout the test period (after the flag leaf stage). The disease severity was evaluated three weeks after *Fusarium* inoculation, and the score was visually determined. The scores ranged from 0 (no infection) to 8 (all infections) as a disease severity to check for 20 spikes per replicate. Deoxynivalenol (DON) ELISA Kit (Nittobo Medical, Japan) was used to measure DON.

### Statistical analysis

Differences in agronomical traits, malting quality, sensory evaluation, DON, and FHB resistance were analyzed using Bell Curve for Excel ver. 4.05 for Windows. Statistical treatments for agronomic traits and malting quality were analyzed using paired-sample t-tests. Sensory scores of the pilot-scale brewing samples and DON were analyzed using independent sample t-tests. The disease severities of FHB infection were analyzed using the Wilcoxon signed-rank test.

## Results

### Agronomic characteristics

**Fig. 2.**
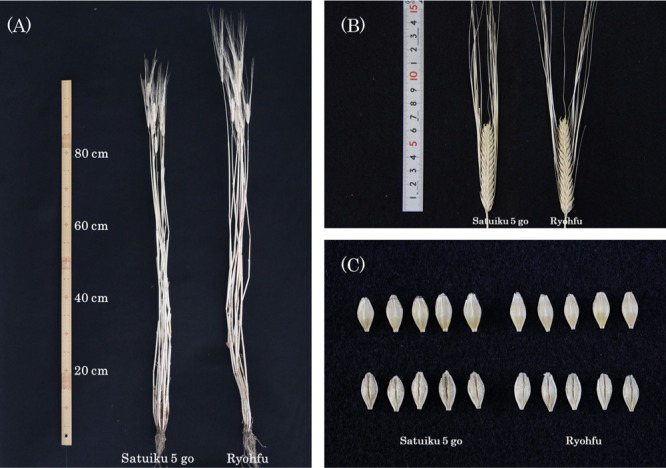
Photographs of (A) standing plants, (B) spikes, (C) grains of ‘Satuiku 5 go’ and ‘Ryohfu’.

Data on the agronomic characteristics in the joint field trials for malting barley were collected from 2016 to 2018. In comparison with ‘Ryohfu’, ‘Satuiku 5 go’ showed 11% higher grain yield (*p* < 0.01; Table 1), earlier heading and maturation date (*p* < 0.01; Table 1), shorter plant height and spike length (*p* < 0.01; Table 1, Fig. 2A), fewer spikes and lower spikelet number (*p* < 0.01; Table 1, Fig. 2B), lower test weight (*p* < 0.01) and thousand kernel weight (*p* < 0.05; Table 1, Fig. 2C) on average over three years.

### FHB resistance

**Fig. 3.**
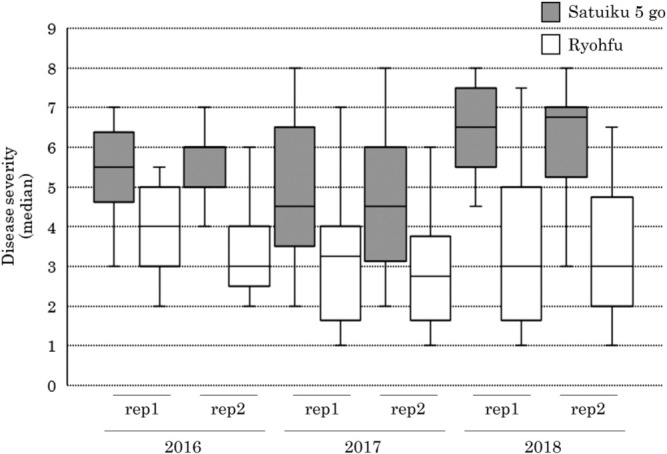
Disease severity of Fusarium head blight. Disease severity: scored 0–8. 0: resistant, 8: susceptible.

The median disease severity of FHB in ‘Satuiku 5 go’ was significantly higher than in ‘Ryohfu’ (Wilcoxon signed-rank test, *p* < 0.05; Fig. 3). This result suggests that ‘Satuiku 5 go’ is more susceptible to FHB compared with ‘Ryohfu’.

### Malting quality

**Fig. 4.**
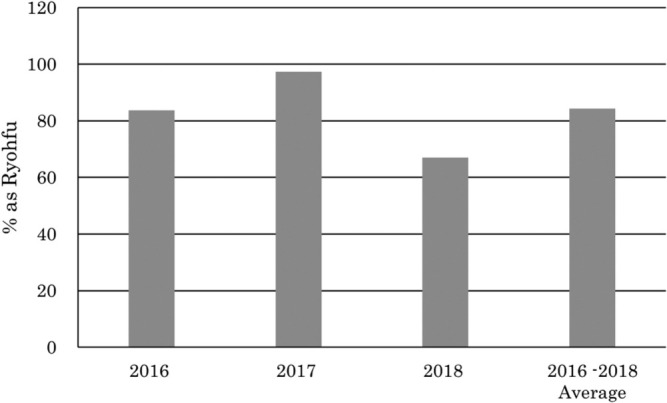
SMM contents of ‘Satuiku 5 go’ in wort compared with that of ‘Ryohfu’.

The diastatic power of ‘Satuiku 5 go’ was approximately 30 points higher than that of ‘Ryohfu’ (*p* < 0.01), and the content of wort β-glucan was lower than that of the control variety (*p* < 0.05; Table 2). The fine extract content of ‘Satuiku 5 go’ was higher than that of ‘Ryohfu’ (*p* < 0.05; Table 2). Furthermore, the soluble nitrogen content of ‘Satuiku 5 go’ was higher than that of ‘Ryohfu’ (*p* < 0.01), but the Kolbach Index, which was calculated as the percentage of soluble nitrogen in total nitrogen, was not significant (Table 2). The SMM of ‘Satuiku 5 go’ in wort tended to be lower than that of ‘Ryohfu’ (Fig. 4).

### Brewing performance in the pilot-scale brewing trial

As observed from malting quality data in the joint field trials for malting barley, ‘Satuiku 5 go’ malt exhibited higher fine extract content, soluble nitrogen content, and diastatic power, and lower wort β-glucan content than the ‘Ryohfu’ malt (Table 3).

**Fig. 5.**
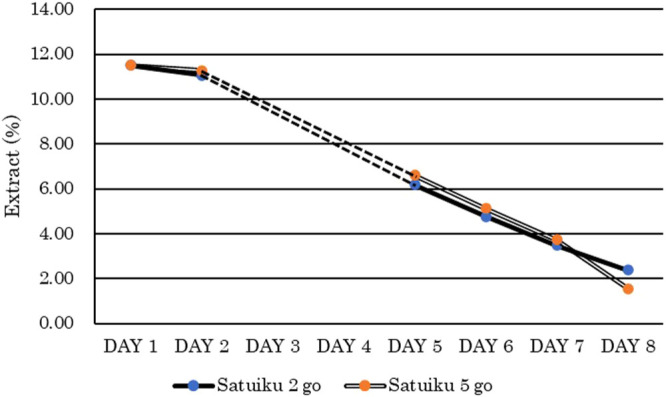
Fermentation Progress of ‘Satuiku 5 go’ and ‘Satuiku 2 go’ in the 100-litter pilot scale brewing trials. The extracts in DAY 3 and DAY 4 were not measured and indicated dashed lines.

During the fermentation process, the extract and number of yeast cells were monitored, and there was no difference between the control and the test varieties (Fig. 5). Wort and beer quality data from the pilot-scale brewing trials are presented in Table 4. No difference was observed between ‘Satuiku 5 go’ beer and ‘Satuiku 2 go’ beer for foam stability, THOD content, or T2N content. Both varieties lacked LOX-1 in barley seeds.

### Sensory evaluation

**Fig. 6.**
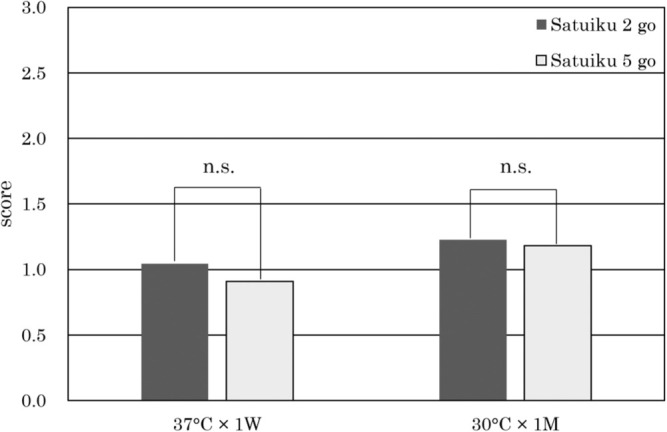
Sensory evaluation of aged beers in the 100-litter pilot scale brewing trials for total staleness. n.s.: not significant at the 5% probability level.

Total staleness in aged beer (37°C for one week and 30°C for one month) is shown in Fig. 6. The sensory scores for ‘Satuiku 5 go’ beer in the pilot-scale brewing trials were slightly lower (meaning “fresher”) than those for ‘Satuiku 2 go’ beer, although the differences were not statistically significant at the 5% probability level.

## Discussion

‘Satuiku 2 go’ was the first LOX-1-less malting barley variety in Japan (Hoki *et al.* 2018) and the commercial production began in Hokkaido in 2019. The yield and malting quality of ‘Satuiku 2 go’ were quite similar to ‘Ryohfu’ which had been a popular variety in Hokkaido and grown over 30 years (Hoki *et al.* 2018). ‘Satuiku 2 go’ was bred by backcrossing ‘Ryohfu’ as the recurrent parent (Hoki *et al.* 2018). We aimed to improve grain yield, malting quality, and brewing quality to maintain malting barley production in Hokkaido, and ‘Satuiku 5 go’ had an 11% higher grain yield than ‘Ryohfu’ (Table 1). The number of spikes of ‘Satuiku 5 go’ was higher than those of ‘Ryohfu’, and the sterile rate of ‘Satuiku 5 go’ was lower than that of ‘Ryohfu’. This suggests that these traits led to higher yields in this variety.

For malting quality, ‘Satuiku 5 go’ tended to have higher soluble nitrogen and fine extract contents than ‘Ryohfu’ (Table 2). Moreover, wort β-glucan content, which affected wort filtration, in ‘Satuiku 5 go’ was significantly lower than in ‘Ryohfu’ (*p* < 0.05), and the diastatic power of ‘Satuiku 5 go’ was significantly higher than that of ‘Ryohfu’ (*p* < 0.01; Table 2). These data indicate that ‘Satuiku 5 go’ had a higher malting quality in terms of the balance of malt modification. The SMM of ‘Hokuiku 39 go’ was 14% higher than that of the control variety, ‘Ryohfu’ in 2005, but that of ‘Satuiku 5 go’ was lower than that of ‘Ryohfu’ in the joint field trials for malting barley in Hokkaido from 2016 to 2018 (Fig. 4).

For brewing performance, both the T2N and THOD contents of ‘Satuiku 5 go’ were similar to those of ‘Satuiku 2 go’, which was a LOX-1-less malting barley variety for Hokkaido in Japan (Table 4). Also, the score of sensory evaluation of both varieties were not significant and the scores were relatively lower (Fig. 6). In the previous report, the beer made from ‘Satuiku 2 go’ showed lower T2N and THOD contents than the beer made from ‘Ryohfu’, which was the recurrent parent of ‘Satuiku 2 go’ and not a LOX-1-less variety, and also showed lower score for beer staleness (Hoki *et al.* 2018). Based on the results of the previous report and the present study, it can be concluded that the LOX-1-less variety ‘Satuiku 5 go’ has capacity of keeping beer freshness similar to that of ‘Satuiku 2 go’ and higher than ‘Ryohfu’.

Based on the results of the FHB resistance from testing 20 spikes in each replication, the FHB disease severity of ‘Satuiku 5 go’ indicated a significantly higher score than that of ‘Ryohfu’ (Wilcoxon signed-rank test, *p* < 0.05; Fig. 3). This result suggests that ‘Satuiku 5 go’ was more susceptible to FHB.

The average of DON content of ‘Satuiku 5 go’ was 0.22 ± 0.072 (average ± standard error) ppm and that of ‘Ryohfu’ was 0.11 ± 0.060 (average ± standard error) ppm in 2018 at five sites. The difference of DON content between ‘Satuiku 5 go’ and ‘Ryohfu’ was not significant even though the disease severity of ‘Satuiku 5 go’ was higher than that of the control variety. *Fusarium graminearum* s. str. was dominant in Hokkaido, and all of *F. graminearum* produced DON (Suga *et al.* 2008). The reason for this discrepancy between the disease severities and DON contents remains unclear. This calls for confirming the DON levels of ‘Satuiku 5 go’ for future tests.

‘Satuiku 5 go’ was LOX-1-less variety and exhibited higher grain yield, higher malting quality, and higher brewing performance. However, the disease severity indicated that it was more susceptible to FHB than ‘Ryohfu’. Malting barley is more susceptible to FHB under hotter and more humid conditions. Therefore, continuous development of novel malting barley varieties that can adapt to climate change is necessary.

## Author Contribution Statement

YT and RK bred the novel varieties. RK created the cross, and YT selected the line for each breeding process. SA, HJ, NA, and RF managed the field trials in Hokkaido. HI and HY developed a method for stable high-yield cultivation of this novel variety. TW conducted the brewing trials and evaluated brewing quality. MK, NH, MN, and TH managed and supervised the breeding programs.

## Acknowledgments

This study was supported by grants from the NARO Bio-Oriented Technology Research Advancement Institution (Research Program on the Development of Innovative Technology). Under the above mentioned project, we formed a consortium with the Hokkaido Research Organization, Tokyo University of Agriculture, and the Hokkaido Malting Barley Federation of Farmers’ Unions. We thank all participating staff and our colleagues at Sapporo Breweries Ltd. for their technical assistance with malting, brewing, and quality analysis.

## Figures and Tables

**Table 1.** Agronomical performance of ‘Satuiku 5 go’ and ‘Ryohfu’ in the joint field trials for malting barley in Hokkaido from 2016 to 2018

Variety	Year	Heading date (M/D)	Maturation date (M/D)	Plant height (cm)	Spike length (cm)	Number of spikes (/m^2^)	Spikelet number (/spike)	Sterile rate (%)	Lodging score*^a^* (0–5)	Grain yield*^b^* (kg/a)	Test weight (g/L)	Grain weight (g/1,000 grains)	Plumpness (%)	Grain yield*^c^* (kg/a)	% as Ryohfu (%)
Ryohfu	2016	6/25	7/31	91.9	6.2	541	21.0	2.5	0.1	47.0	643	47.9	98.1	46.0	100
Satuiku 5 go		6/21	7/30	86.0	5.7	653	19.9	1.8	0.0	50.3	626	47.0	98.5	49.3	107
Ryohfu	2017	6/29	7/29	89.1	5.9	631	20.8	3.4	1.2	38.5	543	43.5	91.7	35.4	100
Satuiku 5 go		6/27	7/28	80.5	5.4	686	19.3	1.3	1.0	44.1	534	42.2	93.5	41.3	116
Ryohfu	2018	6/29	7/30	93.6	5.8	592	20.7	5.6	0.3	40.9	604	46.4	93.4	38.4	100
Satuiku 5 go		6/24	7/29	85.8	5.5	689	19.8	3.1	0.6	44.4	578	45.5	93.8	41.7	111
Ryohfu	2016–2018 avarage	6/28	7/30	91.7	5.9	595	20.8	3.9	0.6	41.4	590	45.7	93.8	39.0	100
Satuiku 5 go		6/25	7/28	84.1	5.5	679	19.6	2.0	0.6	45.7	572	44.6	94.8	43.3	111
t-test*^d^*		**	**	**	**	**	**	*	n.s.	**	**	*	n.s.	**	–

*^a^* 0 = no lodging; 5 = completely lodged *^b^* not screened *^c^* screened by a 2.5 mm sieve *^d^* We conducted a t-test for each trait using 2016–2018 average data. **: 1%; *: 5%; n.s.: not significant at the 5% probability level. 2016 trial grown at three sites (Kamikawa Agr. Exp. Sta.; Kitami Agr. Exp. Sta. and the Tokyo Univ. of Agr.) 2017 trial grown at five sites (Kamikawa Agr. Exp. Sta.; Nakafurano; Kitami Agr. Exp. Sta.; Abashiri and the Tokyo Univ. of Agr.) 2018 trial grown at five sites (Kamikawa Agr. Exp. Sta.; Nakafurano; Kitami Agr. Exp. Sta.; Abashiri and the Tokyo Univ. of Agr.) The average values of each site in each year are shown (n = 13).

**Table 2.** Malting quality profiles of ‘Satuiku 5 go’ and ‘Ryohfu’ in the joint field trials for malting barley in Hokkaido from 2016 to 2018

Variety	Year	Steep-out moisture (%)	Steeping time (hr)	Protein (%)	Fine extract (% d.b.)	Soluble nitrogen (%)	Kolbach Index	Diastatic power (°WK/TN)	Apparent attenuation limit (%)	Wort beta-glucan (mg/L)
Ryohfu	2016	43.2	49.7	10.0	82.9	0.686	43.2	174	86.3	43
Satuiku 5 go		43.0	48.4	10.3	83.1	0.701	42.6	210	86.6	32
Ryohfu	2017	43.1	39.4	11.1	81.0	0.706	40.3	158	86.0	115
Satuiku 5 go		43.1	40.0	10.8	81.5	0.719	41.9	188	87.4	44
Ryohfu	2018	43.1	53.6	10.4	82.3	0.685	41.4	148	85.9	79
Satuiku 5 go		43.1	52.9	10.7	82.4	0.709	41.8	176	86.1	47
Ryohfu	2016–2018 average	43.1	47.2	10.6	81.9	0.693	41.4	158	86.1	84
Satuiku 5 go		43.1	46.9	10.6	82.2	0.711	42.0	189	86.7	43
t-test*^a^*		n.s.	n.s.	n.s.	*	**	n.s.	**	n.s.	*

*^a^* We conducted a t-test for each trait using 2016–2018 average data.**: 1%; *: 5%; n.s.: not significant at the 5% probability level. 2016 trial grown at three sites (Kamikawa Agr. Exp. Sta.; Kitami Agr. Exp. Sta. and the Tokyo Univ. of Agr.) 2017 trial grown at five sites (Kamikawa Agr. Exp. Sta.; Nakafurano; Kitami Agr. Exp. Sta.; Abashiri and the Tokyo Univ. of Agr.) 2018 trial grown at five sites (Kamikawa Agr. Exp. Sta.; Nakafurano; Kitami Agr. Exp. Sta.; Abashiri and the Tokyo Univ. of Agr.) The average values of each site in each year are shown (n = 13).

**Table 3.** Malting quality profiles for 100-litter pilot scale brewing trials

Variety	Steep-out moisture (%)	Malt moisture (%)	Wort color (°EBC)	Apparent attenuation limit (%)	Total protein (%)	Soluble nitrogen (%)	Kolbach Index	Diastatic power (°WK)	Wort beta-glucan (mg/L)
Satuiku 2 go	43.4	7.0	3.9	86.8	10.6	0.804	47.3	320	117
Satuiku 5 go	43.4	6.1	3.7	87.2	11.0	0.793	45.0	336	72

**Table 4.** Brewing performance of ‘Satuiku 5 go’ in the 100-litter pilot scale brewing trials

Variety		Satuiku 2 go	Satuiku 5 go
(*Wort*)			
Extract	(%)	11.68	11.77
BU		42.9	42.7
pH		5.42	5.40
Color	(°EBC)	5.5	5.6
Apparent extract	(%)	1.46	1.47
(*Beer*)			
Original gravity	(%)	11.07	11.03
Apparent extract	(%)	1.35	1.34
Alcohol	(vol.%)	5.12	5.11
Color	(°EBC)	4.0	4.0
BU		21.0	20.4
pH		4.41	4.41
NIBEM	(sec)	213	215
THOD	(mg/L)	2.8	2.2
T2N			
Fresh beer	(μg/L)	0.08	0.09
37°C × 1W	(μg/L)	0.07	0.10
30°C × 1M	(μg/L)	0.09	0.12
